# A high CD8 to FOXP3 ratio in the tumor stroma and expression of PTEN in tumor cells are associated with improved survival in non-metastatic triple-negative breast carcinoma

**DOI:** 10.1186/s12885-021-08636-4

**Published:** 2021-08-06

**Authors:** Monique C. Tavares, Cristina D. Sampaio, Geraldine E. Lima, Victor P. Andrade, Daniel G. Gonçalves, Mariana P. Macedo, Vladmir C. Cordeiro de Lima

**Affiliations:** 1 Department of Medical Oncology, AC Camargo Cancer Center, Rua Professor Antonio Prudente 211, ZIP:01525-001, São Paulo, SP Brazil; 2 Department of Anatomic Pathology, AC Camargo Cancer Center, São Paulo, Brazil; 3 Laboratory of Bioinformatics, International Center for Teaching and Research AC Camargo Cancer Center, São Paulo, Brazil; 4 Departament of Anatomic Pathology, Hospital Sírio Libanês, São Paulo, Brazil

**Keywords:** Triple-negative breast cancer, Immune infiltrate, PTEN, CD8, FOXP3, Survival

## Abstract

**Background:**

Triple-negative mammary carcinoma (TNBC) is an aggressive breast cancer subtype associated with dismal prognosis. The interaction between the immune system and the cancer cells plays a crucial role in tumor development and progression. However, it is still unclear how each diverse cell of the immune system contributes to the prognosis of patients with breast cancer. In this study, we investigated how the cell composition of the immune cell infiltrated modifies the survival of patients with resected TNBC.

**Methods:**

Retrospectively, we collected data from 76 patients diagnosed with non-metastatic TNBC with available tissue blocks for tissue micro-array (TMA) construction. The TMA was constructed using two cores from each tumor block. The expression of CD4, CD8, FOXP3, CD20, CD68, CD163, PD-1, PD-L1, PTEN and phospho-STAT1 was determined by immunohistochemistry.

**Results:**

We observed that the inflammatory infiltrate in TNBC is enriched for M2 macrophages and T lymphocytes (CD4+, CD8+). PD-L1 expression in the stroma was associated with the percentage of TILs (*p* = 0.018) as, PD-L1 expression in the tumor was associated with the percentage of TILs (*p* = 0.049). We found a correlation between TILs and PD-L1 expression in stroma cells (*p* = 0.020) and in tumor cells (*p* = 0.027). In our cohort, we observed a trend for improved survival associated with higher CD8+ (*p* = 0.054) and CD4 +  (*p* = 0.082) cell counts, but the results were not statistically significant. Conversely, the expression of PTEN in tumor cells and a low number of FOXP3+ cells in tumor stroma were both associated with improved OS. The CD8 to FOXP3 ratio and the CD4 to FOXP3 ratio were associated with better OS as well, however, only the CD8 to FOXP3 ratio had its prognostic impact confirmed in the METABRIC TNBC cohort. There was no association between PD-L1 expression and OS.

**Conclusion:**

TNBC tumor microenvironment is enriched for lymphocytes and macrophages. FOXP3 expression and the CD8 to FOXP3 ratio in the tumor stroma as well as the loss of PTEN expression in tumor cells are prognostic factors in non-metastatic TNBC.

**Supplementary Information:**

The online version contains supplementary material available at 10.1186/s12885-021-08636-4.

## Introduction

Triple-negative breast cancer (TNBC) refers to breast carcinomas that lack the expression of hormone receptors (estrogen and progesterone receptors), and that do not express the human epidermal growth factor receptor 2 (HER2) and do not have amplification of the corresponding gene (ERBB2). TNBC corresponds to 15–20% of all newly diagnosed breast cancer [1]. Despite the emergence of new drugs and targeted therapies for the treatment of other breast cancer subtypes, patients with TNBC continue to have a dismal prognosis [2].

The interaction between the immune system cells and cancer cells plays an important role in tumor development and progression and involves a complex interaction of tumor cells with chemical mediators (cytokines and chemokines) and cells of the innate and adaptive immune system [3, 4].

Tumor-infiltrating lymphocytes (TILs) comprise a mixture of cytotoxic T cells, helper T cells, as well as B lymphocytes, macrophages, natural killer (NK) cells, and dendritic cells. Gene expression and cytometry analysis have shown that the percentage of TILs represents an indirect marker of a pre-existing anti-tumor T cell response [5]. TILs are commonly observed at increased levels in TNBC and HER2-positive tumors as compared to estrogen-receptor (ER) positive, HER2-negative tumors [6]. Recently, TILs have been reported to be associated with better prognosis and higher response rates to neoadjuvant therapy in early-stage breast cancer as well as improved response to chemotherapy and trastuzumab [7], besides tumor infiltration by cells expressing CD3, CD8 and CD20 is a potential predictive biomarker of response to chemotherapy [8].

The cytotoxic CD8+ T lymphocytes are crucial components of tumor-specific cellular adaptive immunity which can specifically recognize and kill tumor cells. CD8+ T cells induce tumor cell cytostasis and kill them through cell cycle inhibition, induction of apoptosis, angiostasis, and induction of macrophage tumoricidal activity. In contrast, the FOXP3 positive regulatory T cells (Tregs) effectively suppress the proliferation and the activation of cytotoxic T lymphocytes (CTLs) in a contact-dependent manner or via the release of cytokines such as transforming growth factor-beta [9, 10].

STAT-1 gene is a pleiotropic protein with multiple transcriptional functions. STAT-1 is phosphorylated upon T cell activation by interferon-gamma [11].

Activation of PD-1 by PD-L1 or PD-L2 decreases T cell activity, reduces cytokine production and induces antigen tolerance [12]. Clinical studies with therapies involving the inhibition of these so called immune checkpoints, i.e. the inhibition of co-inhibitory molecules of the immune system (such as PD-1/PD-L1), has demonstrated high tumor response rates in several tumors, and PD-L1 expression is correlated with response to those treatments [13–16]. The loss of PTEN correlates, specifically, with the development of hormone receptor-negative breast cancer, and, in addition to inducing the expression of PD-L1, its loss suppresses the proliferation and survival of T lymphocytes [17].

In our study we evaluated the prognostic value of the different immune cell subtypes and, as well as the association between some immunophenotypic immunohistochemical biomarkers in the inflammatory infiltrate of early triple-negative breast cancer.

## Material and methods

### Population

The study population consisted of all patients diagnosed with non-metastatic (stages I, II and III) TNBC, defined by immunohistochemistry (following the CAP 2010 definition), that had available FFPE tissue blocks, treated at the A. C. Camargo Cancer Center, Brazil, from January 2002 to December 2014. Exclusion criteria were in situ ductal carcinoma and a previous invasive cancer in the last 5 years preceding study entry. The analysis was performed only on biopsies obtained prior to the initiation of any systemic treatment or radiotherapy. Demographics, clinical-pathological characteristics and treatment information were collected from the electronic medical records.

### Analysis of the tumor infiltrating lymphocytes (TILs)

The intensity of the inflammatory infiltrate was evaluated in H&E stained slides using the criteria and recommendations described by Denkert et al. [7] and Loi et al. [18] and standardized by Salgado et al. [19]. All slides were evaluated by two pathologists (V.A.A. and L.G.) with experience in mammary pathology.

### TMA construction

The original slides were revised to confirm histological classification and two representative areas of the tumor were selected to obtain two 1.0 mm cores of each sample, which were transferred from the original tumor block to a receptor block to build the TMA. Areas containing necrosis, hemorrhage, and artifacts were avoided.

### Imumunohistochemical reactions

The expression of CD20 (B lymphocyte B), CD4 and CD8 (T lymphocyte), FOXP3 (regulatory T-lymphocyte-Treg), CD68 (macrophages), CD163 (M2 response macrophages), pSTAT1 and PD-1 was evaluated in inflammatory cells located in the tumor stroma. PD-L1 and PD-L2 expression was evaluated both in the tumor cells and in the inflammatory infiltrate in the tumor stroma. PTEN expression was evaluated in tumor cells (absence of labeling) only.

Immunohistochemical reactions used an automated protocol and ready-to-use reagents and were performed in the Benchmark Ultra (Ventana Medical Systems, Inc.) equipment following standardized dilutions and reaction conditions for each antibody (Table S1).

### PD-L1 expression analysis

PD-L1 expression was evaluated with the SP263 clone ( Spring Bioscience) and was analyzed by direct optical microscopy by a pathologist (M.M.P.) with specialized training. PD-L1 expression was counted as the percentage of cells with positive membrane staining and was evaluated separately in tumor cells and in the stroma. Percentages were registered as 0, 1, 5, 10% and then in increments of 10%. For each sample, the average value obtained from the duplicates was used in the statistical analysis. When only one core was evaluable, the value obtained from this core was employed in the analysis.

### Immune infiltrate analysis

Automated counting of the total number of positive cells in the tumor stroma from each core to each biomarker studied (except for PD-L1) was performed using the Aperio AT2 (Leica Biosystems) equipment and the manufacturer’s suggested protocols for membrane (Aperio Membrane Algorithm) and cytoplasmic (Positive PixelCount v9) staining. The area to be analyzed was manually selected by a pathologist (V.P.A.), without knowledge of the clinical data. In each of the technical replicas in the TMA, an area was delimited, and the number of positive cells for each marker was calculated as cells/mm2. The average of the total number of labeled cells obtained in each of the two cores analyzed for each patient was calculated (for the cases with only one evaluable core, that single core was used, and the cases with loss of the two cores in TMA were excluded from the analysis for that biomarker).

### Statistical analysis

Optimal cutoff points for the biomarkers associated with survival were determined by the maximization of the log-rank test method described by LAUSEN e SCHUMACHER 1992. Association between specific cell population counts and clinical-pathological variables was evaluated by the Chi-squared test or Fisher’s exact test. Correlations were determined by the Spearman’s correlation test. Survival curves were calculated by the Kaplan-Meier method and compared by the log-rank test. All tests were deemed statistically significant when *p* < 0.05.

The definition of the optimal gene expression cutoff for the determination of two groups was calculated by the maximization method of the log-rank *p*-value using the maxstat R package described by Horthorn [20].

## Results

### Patient selection and characteristics

**Fig. 1:**
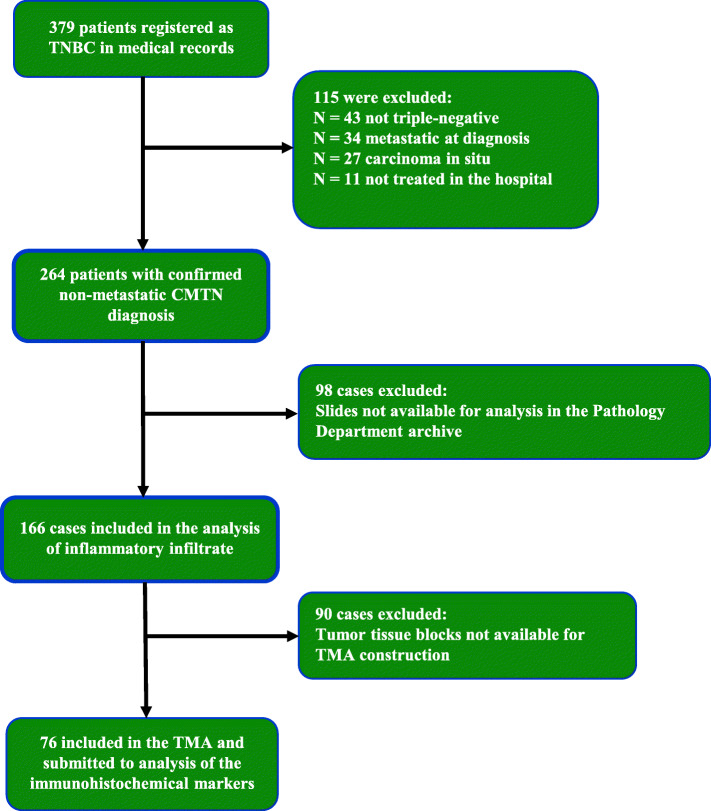
Flowchart of patients included in the study

Initially, 379 patients identified as TNBC in the A. C. Camargo Cancer Center medical records were selected. Of these, 264 fulfilled all inclusion and exclusion criteria from whom clinical and demographic information was collected. From this group, 166 patients with HE slides available in the Pathology Department archive were selected for analysis of TILs, and among them, 76 patients also had paraffin blocks available, which were used to construct the TMA for immunohistochemical analysis (Fig. 1).

### Clinical-pathological features and outcomes of patients included in the TMA analysis

The mean age of the 76 women whose tumors were included in the TMA was 48.4 years old. Twenty-one (27.6%) patients were white and 7 (9.2%) were *BRCA1* mutated. Regarding clinical staging, most patients were diagnosed in stage IIA, 29 (38.2%), or IIB, 14 (18.4%). Seventy-one tumors (93.4%) were of ductal histology and the majority of them was grade 3, (60.5%) 46 patients. Sixty-two (81.6%) received adjuvant treatment and 14 (18.4%) received neoadjuvant chemotherapy. Among the 14 patients who received neoadjuvant treatment, 2 (14.2%) had complete pathologic response, 6 (42.8%) had partial response and 3 (21.4%) had no response. Regarding the clinical outcomes, among the 76 patients analyzed, 18 (23.7%) relapsed, 11 (14.5%) died, 59 (77.6%) had no evidence of disease at the time of the last follow-up, 10 (13.2%) presented disease progression and 7 (9.2%) had loss of follow-up (Table 1).

**Table 1:** Clinical-pathological features and outcomes of patients included in the TMA analysis (*N* = 76)

Variables	Descriptor	Frequency N (%)
**Age**	Mean	48.4
***BRCA*** **mutation**	Wild-type	1 (1.3)
	Mutated	7 (9.2)
	Unknown	68 (89.5)
**Clinical staging**	IA	13 (17.1)
	IB	2 (2.6)
	IIA	29 (38.2)
	IIB	14 (18.4)
	IIIA	7 (9.2)
	IIIB	9 (11.8)
	IIIC	2 (2.7)
**Histology**	IDC	71 (93.4)
	ILC	1 (1.3)
	Metaplastic	4 (5.3)
**Histological grade**	G1	3 (3.9)
	G2	27 (35.6)
	G3	46 (60.5)
**Type of treatment**	Adjuvant	62 (81.6)
	Neoadjuvant	14 (18.4)
**Response to Neoadjuvant chemotherapy**	Complete response	2 (14.2)
	Partial response	6 (42.8)
	Progressive disease	3 (21.4)
	Unknown	3 (21.6)
**Disease recurrence**	Yes	18 (23.7)
	No	57 (75.0)
	Unknown	1 (1.3)
**Disease status**	Disease free	59 (77.6)
	Disease progression	10 (13.2)
	Lost to follow-up	7 (9.2)
**Death due to cancer**	Yes	11 (14.5)
	No	65 (85.5)

### Analysis of the immune cell infiltrate composition

The mean percentage of TILs was 25.78%, and the mean percentage of cells expressing PD-L1 in the tumor and stroma was 14.48 and 20.85%, respectively. We observed that the inflammatory infiltrate in TNBC is enriched for macrophages (cells expressing CD68 and CD163) and T lymphocytes (cells expressing CD8 and CD4), with few cells expressing FOXP3 and pSTAT1 (Table 2). Representative microphotographs for each parameter investigated are depicted in the Supplementary Online Material (Figures S1 to S9).

**Table 2:** Frequency and distribution of TILs and of biomarkers evaluated in the tumor cells or in the tumor stroma

Variable	N^**a**^	Mean cells/mm^**2**^	Standard deviation	% (Mean)
**CD8**	76	1513.53	1353.50	
**FOXP3**	76	26.37	42.87	
**CD20**	76	530.54	1093.47	
**CD4**	76	1288.91	1354.23	
**CD163**	76	2001,41	1630.75	
**CD68**	76	2853.62	2247.43	
**PTEN**	76	119.58	48.47	
**pSTAT1**	76	119.76	197.67	
**PD1**	73	252.59	365.51	
**PD-L1 Tumor**	62			4.53
**PD-L1 Estroma**	48			12.17
**Percentage of TIL**	71			28.34

^a^Number of cases evaluable for the referred parameter

### Association of immune cell subtypes with clinical-pathological characteristics and correlation between biomarkers

PD-L1 expression in the stroma was associated with the percentage of TILs (*p* = 0.018). PD-L1 expression in tumor cells was associated with the percentage of TILs (*p* = 0.049) and with N staging (*p* = 0.046). PD1 expression was associated with histological subtype (*p* = 0.022) (Table 3). The other markers did not show association with any clinical-pathological variable.

**Table 3:**
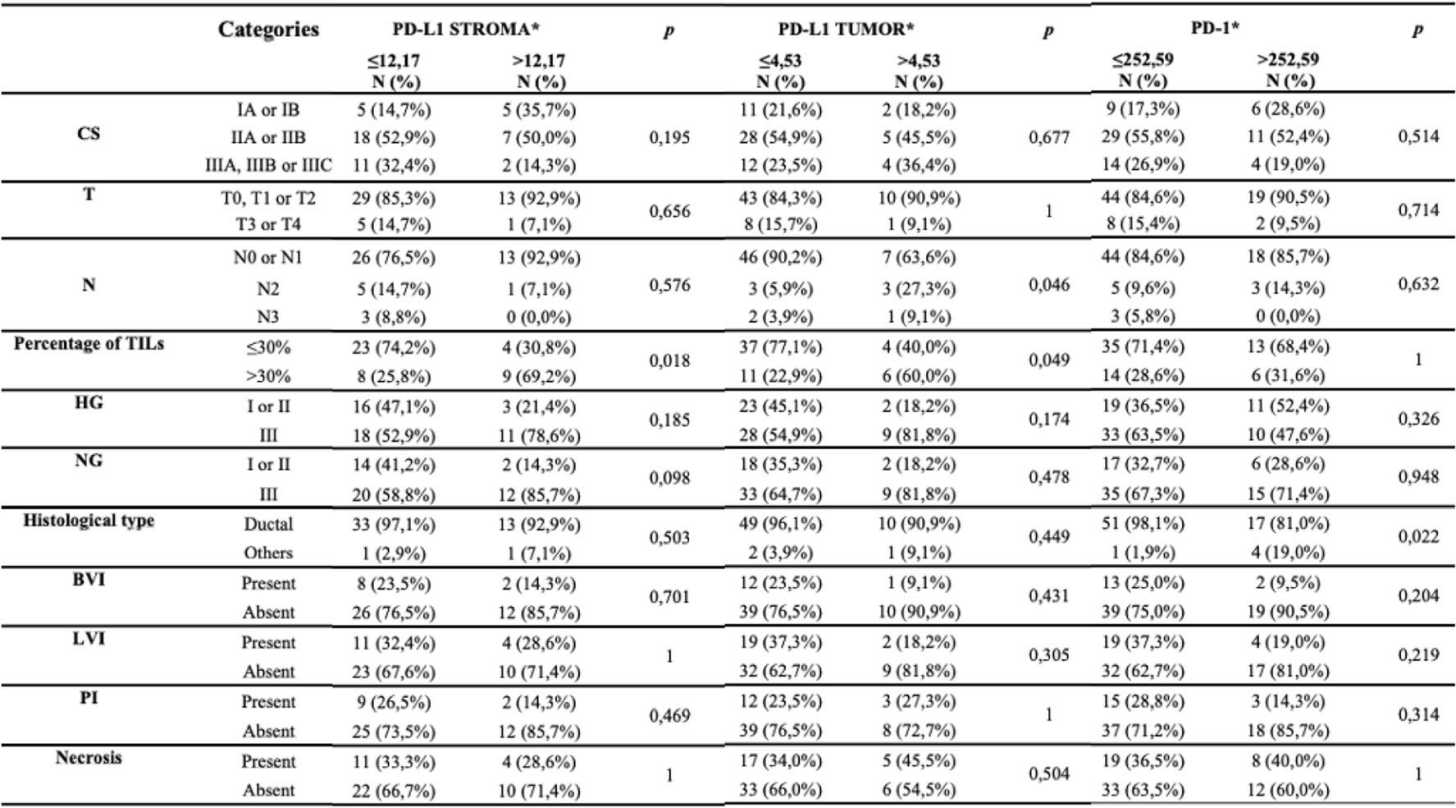
Association between clinical-pathological characteristics and the expression of PD-1, PD-L1 in tumor cells and in stromal cells

*Abbreviations*: *CS* clinical staging, *T* tumor size, *N* regional lymph node status, *HG* histological grade, *NG* nuclear grade, *BVI* blood vascular invasion, *LVI* lymphatic vascular invasion, *PI* perineural invasion

* Median was used to dichotomize the expression of PD-L1 and PD1

We found a positive and statistically significant, although weak, correlation between the percentage of TILs in the stroma and the expression of PD-L1 in stromal cells (*p* = 0.020) and in tumor cells ( = *p* = 0.027).

The following markers also showed a correlation between them: CD4 with CD163, CD8, CD68 and FOXP3; CD163 with CD8, CD68, FOXP3, PD-L1 in the stroma and absence of PTEN expression; CD8 with CD68 and FOXP3; FOXP3 with absence of PTEN expression; CD68 with FOXP3 and absence of PTEN expression; and stromal PDL-1 with tumor PD-L1 and absence of PTEN expression. The number of FOXP3 positive cells were also inversely correlated with the expression of PD-L1 in the tumor (Table 4).

**Table 4:** Correlation of the percentage of TILs and the expression of the studied biomarkers among themselves. Correlations were calculated by Spearman’s test. Statistically significant associations are highlighted in bold. The ρ (rho) indicates the strength of the correlation and was classified as follows: *ρ* = 0.00 to 0.019 (very weak correlation), *ρ* = 0.20 to 0.39 (weak correlation), *ρ* = 0, 40 to 0.69 (moderate correlation), *ρ* = 0.70 to 0.89 (strong correlation), *ρ* = 0.90 to 1.00 (very strong correlation)

Markers	CD20	CD4	CD163	CD8	CD68	FOXP3	PD-L1 Stroma	PD-L1 Tumor	PD1	PTEN Absent	pSTAT1
TIL*											
ρ	0.227	0.143	0.214	0.209	0.163	−0.027	**0.350**	**0.290**	0.139	0.072	0.142
*p-value*	0.057	0.234	0.073	0.080	0.176	0.825	**0.020**	**0.027**	0.259	0.550	0.236
**CD20**											
ρ		0.049	0.010	−0.001	− 0.141	− 0.021	0.210	0.213	−0.105	0.010	−0.140
*p-value*		0.677	0.933	0.990	0.226	0.858	0.153	0.097	0.378	0.930	0.227
**CD4**											
ρ			**0.419**	**0.658**	**0.791**	**0.52**	0.130	0.0003	0.046	0.207	0.062
*p-value*			**0.0002**	**< 0.0001**	**< 0.0001**	**< 0.0001**	0.380	0.998	0.699	0.073	0.595
**CD163**											
ρ				**0.263**	**0.544**	**0.310**	**0.459**	0.234	0.169	**0.410**	0.070
*p-value*				**0.022**	**< 0.0001**	**0.006**	**0.001**	0.067	0.152	**< 0.0001**	0.551
**CD8**											
ρ					**0.604**	**0.374**	0.084	−0.041	−0.031	0.056	0.096
*p-value*					**0.001**	**0.001**	0.569	0.753	0.794	0.633	0.410
**CD68**											
**ρ**						**0.517**	0.087	−0.061	0.087	**0.249**	0.029
*p-value*						**< 0.0001**	0.555	0.638	0.465	**0.030**	0.802
**FOXP3**											
**ρ**							0.181	**−0.278**	0.033	**0.281**	0.174
*p-value*							0.218	**0.029**	0.780	**0.014**	0.133
**PD-L1 Stroma**											
**ρ**								**0.519**	0.188	**0.451**	−0.087
*p-value*								**< 0.0001**	0.206	**0.001**	0.557
**PD-L1 Tumor**											
**ρ**									0.110	0.187	−0.031
*p-value*									0.399	0.145	0.811
**PD-1**											
**ρ**										0.042	−0.153
*p-value*										0.726	0.198
**PTEN**											
**ρ**											0,004
*p-value*											0.972

### Impact of biomarker expression on overall survival

**Fig. 2:**
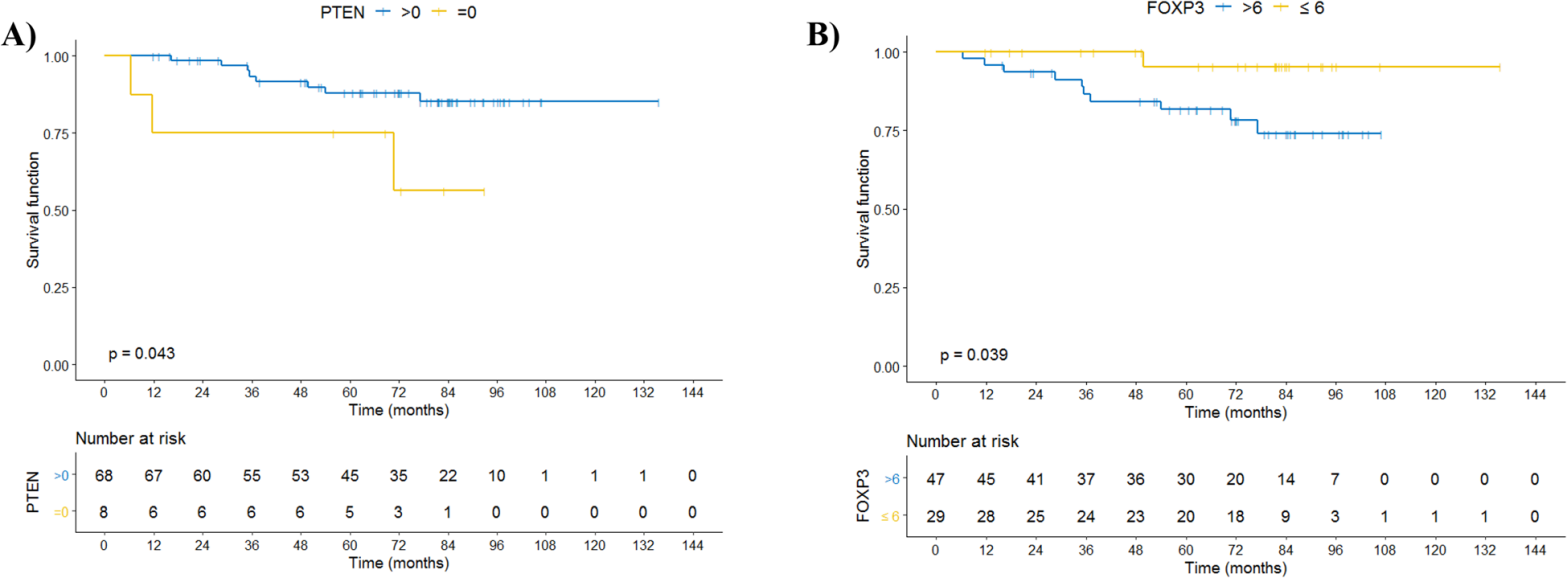
Overall survival (OS) according to FOXP3 and PTEN expression. 2A) OS according FOXP3 expression in stromal cells. 2B) OS according to PTEN (absence of expression) in tumor cells. Survival curves were calculated by the Kaplan-Meier method and compared with the log-rank test

**Fig. 3:**
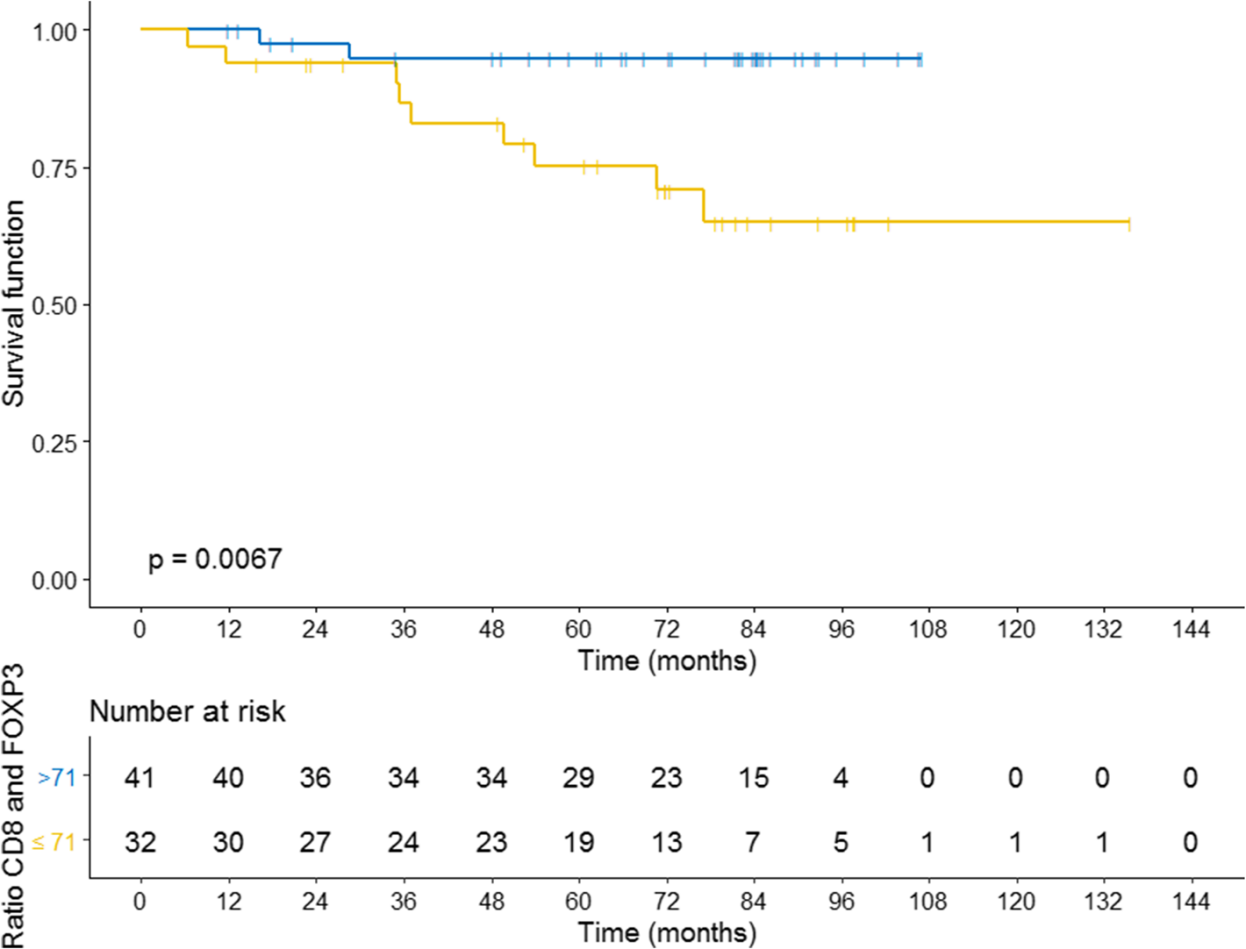
Overall survival curves according to the ratio between the number of CD8 and FOXP3 positive cells. Survival curves were calculated by the Kaplan-Meier method and compared by the log-rank test

The variables associated with overall survival were the lack of expression of PTEN in tumor cells and the number of cells positive for FOXP3 (Fig. 2a and b). The ratio between CD8 and FOXP3 (Fig. 3) and between CD4 and FOXP3 were associated with improved OS (Figure S14).

Although the median survival was numerically different (77 and 120 months, respectively for PD-L1 expression in tumor> 1% and ≤ 1%; *p* = 0.224) for patients whose tumors had high PD-L1 expression, this was not statistically significant (Figure S10). Similarly, PD-L1 expression in the stroma and the number of cells positive for CD8, CD4, CD20, pSTAT-1, CD68, CD163 or PD-1 in the stroma were not associated with overall survival (Figures S11 to S13 and S15 to S19).

### Impact of CD8A to FOXP3 ratio on the survival of TNBC in the METABRIC cohort

**Fig. 4:**
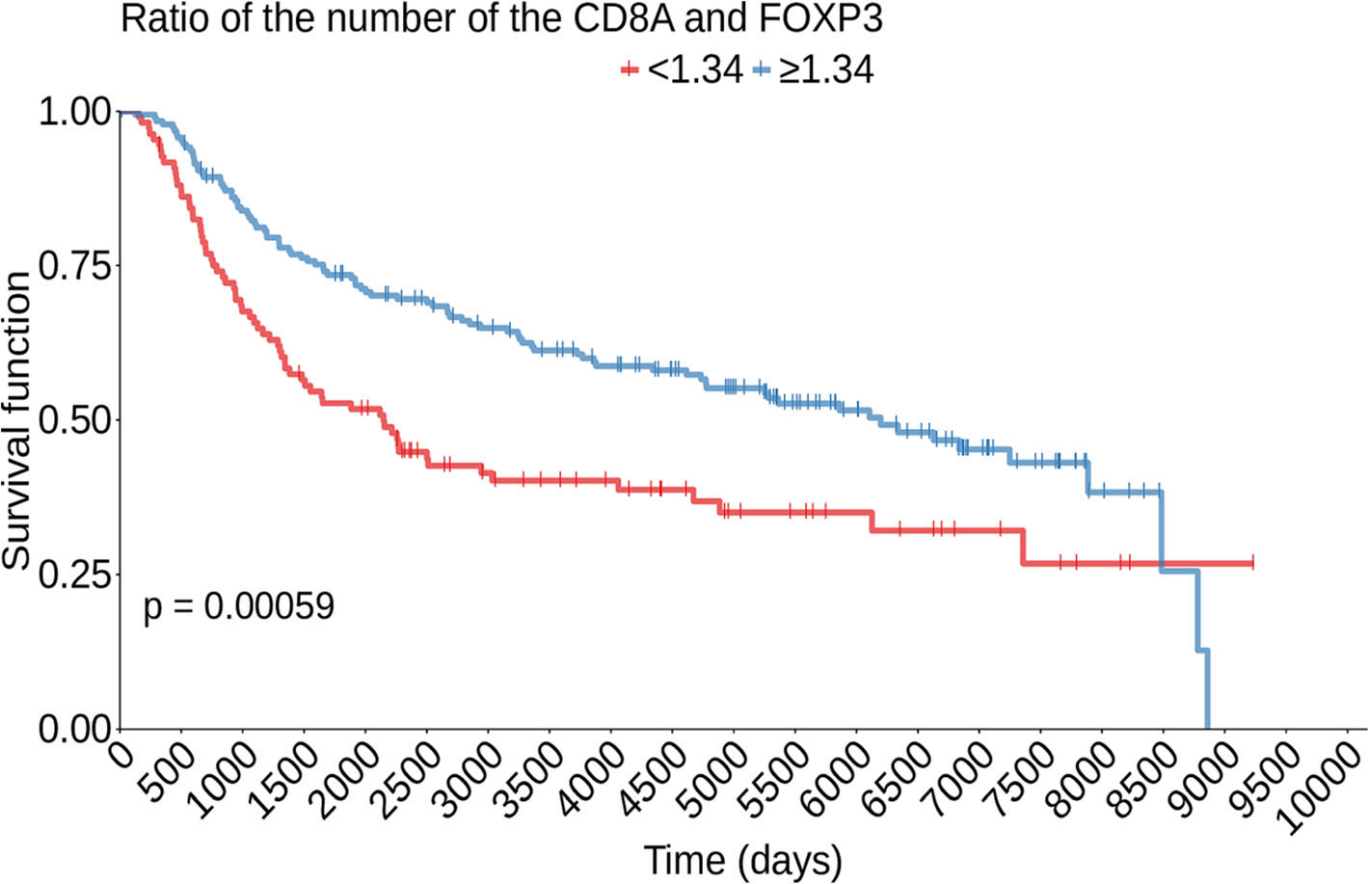
Overall Survival of triple-negative breast cancer patients in the METABRIC cohort according to the ratio between the expression of CD8A and FOXP3. The groups were stratified using optimal cut-off derived by the max-stat method. Patients with the ratio between *CD8A* and *FOXP3* expression higher than or equal to 1.34 had better overall survival. Survival curves were calculated using the Kaplan-Meier method and compared by the log-rank test

We calculated the overall survival of non-metastatic, triple-negative breast cancer patients included in the METABRIC according to the ratio between the expression of CD8A and FOXP3 genes (CD8A/FOXP3 ratio). Similar to what was observed in our cohort, patients whose tumors had a high CD8A to FOXP3 ratio had improved overall survival (Fig. 4). We also evaluated the prognostic impact of *FOXP3* gene expression, and of *CD4* to *FOXP3* gene expression ratio. *FOXP3* expression maintained its association with OS (Figure S20), however we could not confirm the prognostic impact of *CD4/FOXP3* ratio (Figure S21). We could not validate the prognostic impact of PTEN in the METABRIC dataset since the gene was not annotated.

## Discussion

In our TNBC cohort, the immune/inflammatory infiltrate observed in tumor samples was enriched with macrophages (mainly with M2 phenotype) and T lymphocytes. Although we observed a high number of CD8 + and CD4 + cells in the stroma of TNBC, these cells were directly correlated with increased numbers of CD68 + and CD163 + cells, as well as FOXP3 + cells. Besides, the number of CD8 + cells also correlated with a higher number of PD-L1+ cells in the stroma. We hypothesize that, although TN breast tumors may effectively attract T lymphocytes at some point in their progression, these lymphocytes probably have their effector activity suppressed by the co-recruitment of immunosuppressive cells (M2 macrophages and regulatory lymphocytes) and the acquisition of an exhausted phenotype due to the PD-L1 expression, nevertheless this needs to be further validated.

The description of the frequency of these cells in the intratumoral or stromal compartments is very heterogeneous in the literature, as well as the determination of its clinical significance [21]. The evaluation of the intratumoral cytokine profile and a more detailed evaluation of the immunophenotype of the cells in the tumor immune infiltrate by flow or mass cytometry would be very informative.

We also found a positive and statistically significant correlation between the percentage of cells expressing PD-L1 in the stroma (*p* = 0.020) and PD-L1 in the tumor (*p* = 0.027), reinforcing some data from the literature suggesting that the higher the inflammatory tumor infiltrate the higher the expression levels of PD-L1 [22]. Nevertheless, PD-L1 expression, both in the stroma and in the tumor was not associated with OS. Muenst et al. [17] demonstrated that PD-L1 expression is a negative prognostic factor for OS, independent of the histological tumor subtype. However, other recent studies have showed contradictory results [15, 23].

In some samples analyzed for PD-L1 expression, both cores in the TMA were unevaluable because the tissue detached from the slide, in other cases, the core represented a tumor with high cellularity and did not contain stroma. This might have somehow influenced the results of association tests with clinical-pathological characteristics, since this can provoke an imbalance in the proportion of informative cases in each scenario. On the other hand, we believe the correlation tests were not affected since they involve only samples that are informative for both markers in that correlation.

Lymphocytes infiltrated into the tumor are an important immune component of the cancer response. Denardo et al. [24] analyzed the density of CD4, CD8 and CD68 leukocytes in tumor tissues obtained at the time of primary surgery from 179 patients with breast cancer who were treatment naive. They observed that a high density of CD4+ T cells and low density of CD8+ T cells correlated with reduced OS, while CD68+ cell density was not associated with OS. However, there was an inverse correlation between stromal infiltration by CD68+ macrophages and CD8+ T lymphocytes [24].

According to Sasha et al. [25], the number of CD8+ T cells is a predictor of better clinical outcome. Intratumoral infiltration by CD8+ T cells was associated with longer cancer-specific survival (HR 0.55, 95% CI, 0.39–0.78, *p* = 0.001) [25]. In our cohort, we observed a trend for improved survival associated with a higher CD8+ (*p* = 0.054) and CD4 + (*p* = 0.082) cell counts, but the results were not statistically significant.

The only variables associated with overall survival were the lack of PTEN expression in tumor cells and the number of FOXP3+ cells in the stroma. Since the optimal cut-points for OS evaluation of these variables were not associated with any clinical variable, we believe that these can be independent markers of OS, but, given the small sample size, we could not perform a multivariate analysis to check this hypothesis.

However, as we analyzed the association of OS with the ratio between the number of CD8+ and FOXP3+ cells (*p* = 0.007) and of CD4+ and FOXP3+ cells (*p* = 0.034), we found that a higher relative proportion of CD8+ or CD4+ lymphocytes in relation to FOXP3+ lymphocytes was associated with improved OS. Together, these data suggest that the T lymphocytes in the tumor microenvironment may exhibit antitumor activity and promote growth control, as opposed to events favoring immunosuppression (regulatory lymphocyte infiltration). This data is replicated in the TNBC cohort of METABRIC for those patients whose tumors have a high CD8A/FOXP3 ratio.

Liu et al. [9] studied the association between T cells (Treg) and CD8+ cytotoxic T lymphocytes (CTLs) with patient survival, histopathologic features, and molecular subtypes in 1270 cases of invasive breast carcinoma. They showed that an increased infiltration by Tregs and CTLs inside the tumors was associated with unfavorable characteristics, like high histologic grade and negative ER and PR status. In addition, high Treg infiltration was also associated with decreased overall survival (OS) and progression-free survival (PFS). On the other hand, a high CTL/Treg ratio in the tissue surrounding the tumor was significantly associated with improved OS and PFS [9]. Miyan et al. [25] evaluated the expression of CD8, FOXP3, and CD3 in 177 patients with primary, invasive, unilateral early-stage breast cancer of all molecular subtypes and observed that T-cell infiltration was associated with hormone receptor negative tumors, high proliferation rate, high histological grade, and with large tumors. Basal-like tumors had the highest number of FOXP3+ T-cells, with an unfavorable ratio to cytotoxic CD8 + .

**Fig. 5:**
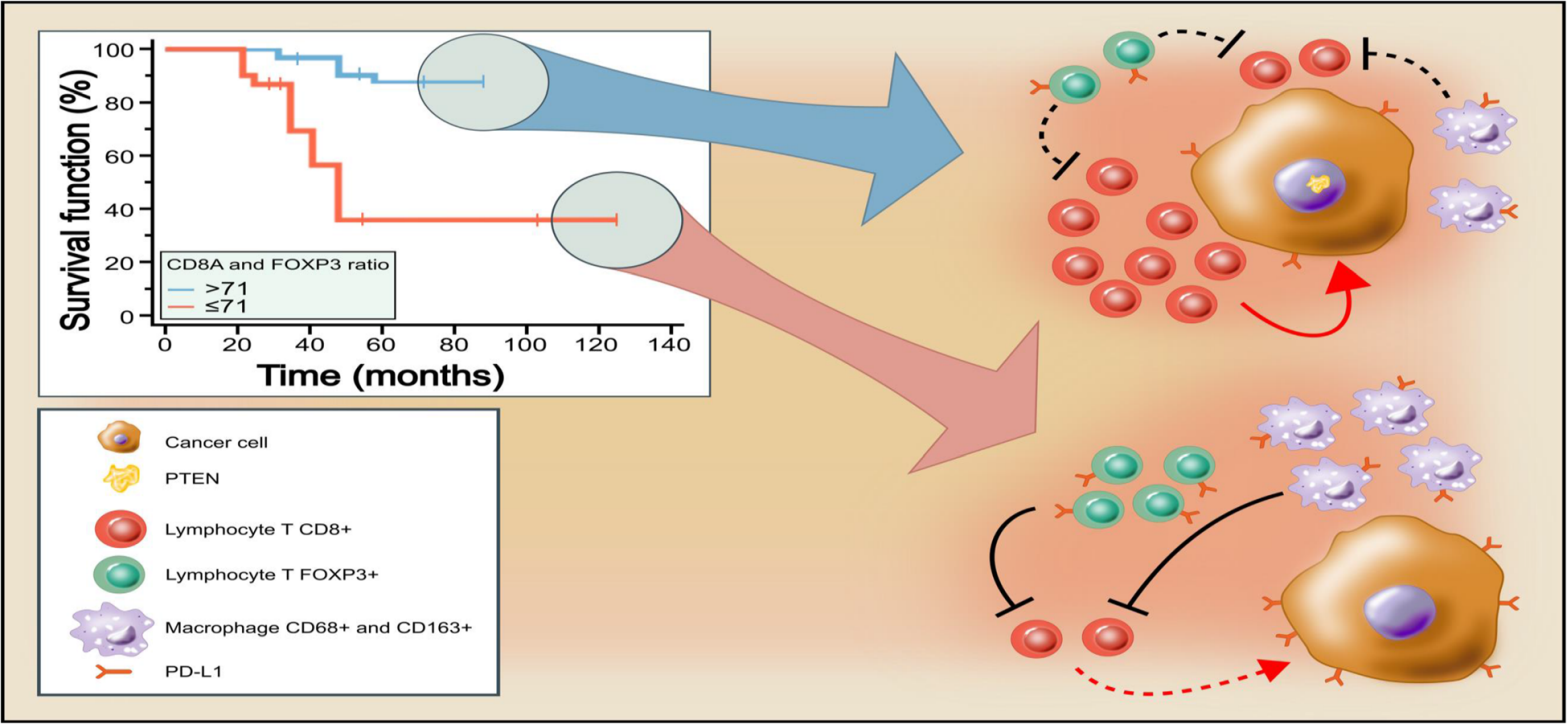
The tumor immune microenvironment in CMTN is associated with overall survival. A more exuberant mononuclear infiltrate is associated with increased overall survival, as well as with increased PD-L1 expression in both the stroma and tumor cells. As that characteristic (PD-L1 expression) was also associated with infiltration by CD8 expressing cells, we believe that those tumors (upper group in the figure) represent a more immunogenic subgroup which actively recruits the adaptive immune system, however, at the same time, as a mechanism of adaptive resistance, it triggers the expression of PD-L1 (exhaustion). Effective anti-tumor activity depends on the ratio between cells expressing CD8 (cytotoxic T lymphocytes) and FOXP3 (regulatory lymphocytes). Besides, loss of PTEN expression was also associated with poorer survival and infiltration by macrophages (cells expressing CD68 and CD163) and regulatory lymphocytes. The red arrows represent the likely final effect on the tumor. Barred arrows indicate inhibitory effect. Tipped arrows indicate anti-tumor activity

Our data suggest that the presence of exuberant inflammatory infiltrate in TNBC is associated with PD-L1 expression both in the tumor itself and in the tumor stroma, as well as with enrichment for T lymphocytes (CD8+ and CD4+), which may make these tumors important targets for treatment with PD-1 or PD-L1 inhibitors. Furthermore, macrophages and regulatory T lymphocytes probably represent important counter-regulatory mechanisms that the tumor recruits to allow its escape of the adaptive immune response and a high CD8 to FOXP3 ratio in the tumor stroma is a predictor of improved survival in non-metastatic TNBC (Fig. 5). In the future, it would be interesting to evaluate the role of strategies that deplete FOXP3+ cells in the tumor microenvironment.

## Supplementary Information

**Additional file 1 **: **Table S1**. Characteristics of antibodies used and standardization of immunohistochemical reactions. **Figure S1**. Microscopic analysis of the tumor infiltrating lymphocytes. A - Triple-negative breast carcinoma rich in lymphocytes. B - Triple-negative breast carcinoma without peritumoral lymphocytes. H&E staining. 100x magnification. **Figure S2**. PD-L1 expression on stromal and tumor cells. A - PD-L1 membrane staining in stromal cells (macrophages). 400x magnification. B - PD-L1 membrane staining in tumor cells. Brown coloration indicates PD-L1 expression (immunohistochemistry with DAB). 400x magnification. **Figure S3**. PTEN expression in tumor cells. Strong PTEN cytoplasmic expression in tumor cells. Brown coloration indicates PTEN expression (immunohistochemistry with DAB). 100x magnification. **Figure S4**. CD8 expression in stromal cells. Moderate infiltrate by CD8 lymphocytes at tumor board. Brown coloration indicates CD8 expression (immunohistochemistry with DAB). 100x magnification. **Figure S5**. CD163 expression in stromal cells. Macrophages expressing cytoplasmic CD163 with a granular pattern. Brown coloration indicates CD163 expression (immunohistochemistry with DAB). 100x magnification. **Figure S6**. CD68 expression in stromal cells. Dense macrophagic infiltration with cytoplasmic CD68 expression on the right core. The core on the left side shows a few cells expressing CD68. Brown coloration indicates CD68 expression (immunohistochemistry with DAB). 100x magnification. **Figure S7**. CD20 expression in stromal cells. Lymphocytes with strong membranal CD20 expression in tumor stroma. Brown coloration indicates CD20 expression (immunohistochemistry with DAB). 100x magnification. **Figure S8**. FOXP3 expression in stromal cells. Rare lymphocytes with FOXP3 nuclear expression. Brown coloration indicates CD20 expression (immunohistochemistry with DAB). 400x magnification. **Figure S9**. CD4 expression in stromal cells. Intense infiltrate by CD4 lymphocytes at tumor board. Brown coloration indicates CD4 expression (immunohistochemistry with DAB). 100x magnification. **Figure S10.** Overall survival curves according to labeling for PD-L1 in the stroma (> 5% or ≤ 5%). Survival curves were calculated using the Kaplan-Meier method and compared by the log-rank method. The vertical strokes in the curves represent censored cases. **Figure S11**. Overall survival curves according to PD-L1 expression in tumor cells. Survival curves were calculated by the Kaplan-Meier method and compared by the log-rank test. **Figure S12**. Overall survival curves according to the number of CD8 positive cells. Survival curves were calculated by the Kaplan-Meier method and compared by the log-rank test. **Figure S13**. Overall survival curves according to the number of CD4 positive cells. Survival curves were calculated using the Kaplan-Meier method and compared by the log-rank method. The vertical strokes in the curves represent censored cases. **Figure S14**. Overall survival curves according to the ratio of the number of CD4 and FOXP3 positive cells. Survival curves were calculated using the Kaplan-Meier method and compared by the log-rank method. The vertical strokes in the curves represent censored cases. **Figure S15**. Overall survival curves according to the number of CD20 positive cells. Survival curves were calculated using the Kaplan-Meier method and compared by the log-rank method. The vertical strokes in the curves represent censored cases. **Figure S16**. Overall survival curves according to the number of pSTAT1 positive cells. Survival curves were calculated using the Kaplan-Meier method and compared by the log-rank method. The vertical strokes in the curves represent censored cases. **Figure S17**. Overall survival curves according to the number of CD68 positive cells. Survival curves were calculated using the Kaplan-Meier method and compared by the log-rank method. The vertical strokes in the curves represent censored cases. **Figure S18**. Overall survival curves according to the number of CD163 positive cells. Survival curves were calculated using the Kaplan-Meier method and compared by the log-rank method. The vertical strokes in the curves represent censored cases. **Figure S19**. Overall survival curves according to the number of PD-1 positive cells. Survival curves were calculated using the Kaplan-Meier method and compared by the log-rank method. The vertical strokes in the curves represent censored cases. **Figure S20.** Overall Survival of triple-negative breast cancer patients in the METABRIC cohort according to the expression of *FOXP3*. The groups were stratified using optimal cut-off derived by the max-stat method. Survival curves were calculated using the Kaplan-Meier method and compared by the log-rank test. **Figure S21.** Overall Survival of triple-negative breast cancer patients in the METABRIC cohort according to the ratio between the expression of *CD4* to *FOXP3*. The groups were stratified using optimal cut-off derived by the max-stat method. Survival curves were calculated using the Kaplan-Meier method and compared by the log-rank test.

## Data Availability

Further information is available on request. This work was the result of the Master’s degree dissertation of the principal author, Monique Celeste Tavares. All data generated or analyzed during this study are included in this published article and in its supplementary files.
