# Supplementary material for: A high CD8 to FOXP3 ratio in the tumor stroma and expression of PTEN in tumor cells are associated with improved survival in non-metastatic triple-negative breast carcinoma
Source: BMC Cancer. 2021 Aug 6;21:901. doi: 10.1186/s12885-021-08636-4 (PMC8343973; doi:10.1186/s12885-021-08636-4)
Supplement: Supplementary file 1 — Additional file 1 : Table S1. Characteristics of antibodies used and standardization of immunohistochemical reactions. Figure S1. Microscopic analysis of the tumor infiltrating lymphocytes. A - Triple-negative breast carcinoma rich in lymphocytes. B - Triple-negative breast carcinoma without peritumoral lymphocytes. H&E staining. 100x magnification. Figure S2. PD-L1 expression on stromal and tumor cells. A - PD-L1 membrane staining in stromal cells (macrophages). 400x magnification. B - PD-L1 membrane staining in tumor cells. Brown coloration indicates PD-L1 expression (immunohistochemistry with DAB). 400x magnification. Figure S3. PTEN expression in tumor cells. Strong PTEN cytoplasmic expression in tumor cells. Brown coloration indicates PTEN expression (immunohistochemistry with DAB). 100x magnification. Figure S4. CD8 expression in stromal cells. Moderate infiltrate by CD8 lymphocytes at tumor board. Brown coloration indicates CD8 expression (immunohistochemistry with DAB). 100x magnification. Figure S5. CD163 expression in stromal cells. Macrophages expressing cytoplasmic CD163 with a granular pattern. Brown coloration indicates CD163 expression (immunohistochemistry with DAB). 100x magnification. Figure S6. CD68 expression in stromal cells. Dense macrophagic infiltration with cytoplasmic CD68 expression on the right core. The core on the left side shows a few cells expressing CD68. Brown coloration indicates CD68 expression (immunohistochemistry with DAB). 100x magnification. Figure S7. CD20 expression in stromal cells. Lymphocytes with strong membranal CD20 expression in tumor stroma. Brown coloration indicates CD20 expression (immunohistochemistry with DAB). 100x magnification. Figure S8. FOXP3 expression in stromal cells. Rare lymphocytes with FOXP3 nuclear expression. Brown coloration indicates CD20 expression (immunohistochemistry with DAB). 400x magnification. Figure S9. CD4 expression in stromal cells. Intense infiltrate by CD4 lymphocytes at tumor boar [file 12885_2021_8636_MOESM1_ESM.docx]

**Supplementary Online Material**

**Table S1. Characteristics of antibodies used and standardization of immunohistochemical reactions.**

| **Target** | **Manufacturer** | **Clone** | **Catalog**  **Number** | **Standardization**  **(equipment / dilution / antigen retrieval time + type of solution / antibody incubation time)** | **Staining pattern** | **Positive control** |
| --- | --- | --- | --- | --- | --- | --- |
| **PD-L1/ CD274** | Spring Bioscience | SP263 | M442S | Ventana; 1/50  36CC2/56Ab | Membrane | Placenta |
| **CD163** | Vector Laboratories | 10D6 | VP-C374 | Ventana; 1/50  30CC1/32Ab | Membrane | Spleen |
| **PD1** | Abcam | NAT105 | ab52587 | Ventana; 1/50  30CC1/32Ab | Membrane | Tonsil |
| **FOXP3** | Abcam | 236A/ E7 | ab20034 | Ventana; 1/5  30CC1/32Ab | Nucleus | Tonsil |
| **pSTAT1 (Tyr701)** | Cell Signaling | 58D6 | #9167 | Ventana; 1/100  30CC1/32Ab | Nucleus | Non-Hodgkin's Lymphoma |
| **PTEN** | Thermo Scientific | 17-A | MS-1601 | Ventana; 1/50  60CC1/48Ab | Cytoplasm | Stomach |
| **CD4** | Ventana | SP35 | 790-4423 | Ventana; pre-diluted  30CC1/32Ab | Membrane | Tonsil |
| **CD8** | Ventana | SP57 | 790-4460 | Ventana; pre-diluted  30CC1/32Ab | Membrane | Tonsil |
| **CD20** | Ventana | L26 | 760-2531 | Ventana; pre-diluted  30CC1/32Ab | Membrane | Tonsil |
| **CD68** | Ventana | KP-1 | 790-2931 | Ventana; pre-diluted  30CC1/32Ab | Cytoplasm  Membrane | Tonsil |

**A**

**B**

**Figure S1 - Microscopic analysis of the tumor infiltrating lymphocytes.** A - Triple-negative breast carcinoma rich in lymphocytes. B - Triple-negative breast carcinoma without peritumoral lymphocytes. H&E staining. 100x magnification.

**A**

**B**

**Figure S2 - PD-L1 expression on stromal and tumor cells.** A - PD-L1 membrane staining in stromal cells (macrophages). 400x magnification. B - PD-L1 membrane staining in tumor cells. Brown coloration indicates PD-L1 expression (immunohistochemistry with DAB). 400x magnification.

**Figure S3 – PTEN expression in tumor cells.** Strong PTEN cytoplasmic expression in tumor cells. Brown coloration indicates PTEN expression (immunohistochemistry with DAB). 100x magnification.

**Figure S4 – CD8 expression in stromal cells.** Moderate infiltrate by CD8 lymphocytes at tumor board. Brown coloration indicates CD8 expression (immunohistochemistry with DAB). 100x magnification.

**Figure S5 – CD163 expression in stromal cells.** Macrophages expressing cytoplasmic CD163 with a granular pattern. Brown coloration indicates CD163 expression (immunohistochemistry with DAB). 100x magnification.

**Figure S6 – CD68 expression in stromal cells.** Dense macrophagic infiltration with cytoplasmic CD68 expression on the right core. The core on the left side shows a few cells expressing CD68. Brown coloration indicates CD68 expression (immunohistochemistry with DAB). 100x magnification.

**Figure S7 – CD20 expression in stromal cells.** Lymphocytes with strong membranal CD20 expression in tumor stroma. Brown coloration indicates CD20 expression (immunohistochemistry with DAB). 100x magnification.

**Figure S8 – FOXP3 expression in stromal cells.** Rare lymphocytes with FOXP3 nuclear expression. Brown coloration indicates CD20 expression (immunohistochemistry with DAB). 400x magnification.

**Figure S9 – CD4 expression in stromal cells.** Intense infiltrate by CD4 lymphocytes at tumor board. Brown coloration indicates CD4 expression (immunohistochemistry with DAB). 100x magnification.

**Figure S10** **- Overall survival curves according to labeling for PD-L1 in the stroma (> 5% or ≤5%).** Survival curves were calculated using the Kaplan-Meier method and compared by the log-rank method. The vertical strokes in the curves represent censored cases.

**Figure S11 -** **Overall survival curves according to PD-L1 expression in tumor cells.** Survival curves were calculated by the Kaplan-Meier method and compared by the log-rank test.

**Figure S12** **- Overall survival curves according to the number of CD8 positive cells.** Survival curves were calculated by the Kaplan-Meier method and compared by the log-rank test.

**Figure S13** - **Overall survival curves according to the number of CD4 positive cells.** Survival curves were calculated using the Kaplan-Meier method and compared by the log-rank method. The vertical strokes in the curves represent censored cases.

**Figure S14 - Overall survival curves according to the ratio of the number of CD4 and FOXP3 positive cells.** Survival curves were calculated using the Kaplan-Meier method and compared by the log-rank method. The vertical strokes in the curves represent censored cases.

**Figure S15 - Overall survival curves according to the number of CD20 positive cells.** Survival curves were calculated using the Kaplan-Meier method and compared by the log-rank method. The vertical strokes in the curves represent censored cases.

**Figure S16 - Overall survival curves according to the number of pSTAT1 positive cells.** Survival curves were calculated using the Kaplan-Meier method and compared by the log-rank method. The vertical strokes in the curves represent censored cases.

**Figure S17** **- Overall survival curves according to the number of CD68 positive cells.** Survival curves were calculated using the Kaplan-Meier method and compared by the log-rank method. The vertical strokes in the curves represent censored cases.

**Figure S18 - Overall survival curves according to the number of CD163 positive cells.** Survival curves were calculated using the Kaplan-Meier method and compared by the log-rank method. The vertical strokes in the curves represent censored cases.

 **Figure S19** **- Overall survival curves according to the number of PD-1 positive cells.** Survival curves were calculated using the Kaplan-Meier method and compared by the log-rank method. The vertical strokes in the curves represent censored cases.

**Figure S20. Overall Survival of triple-negative breast cancer patients in the METABRIC cohort according to the expression of *FOXP3*.** The groups were stratified using optimal cut-off derived by the max-stat method. Survival curves were calculated using the Kaplan-Meier method and compared by the log-rank test.

**Figure S21. Overall Survival of triple-negative breast cancer patients in the METABRIC cohort according to the ratio between the expression of *CD4* to *FOXP3*.** The groups were stratified using optimal cut-off derived by the max-stat method. Survival curves were calculated using the Kaplan-Meier method and compared by the log-rank test.
